# Association of Suicide and Other Mortality With Emergency Department Presentation

**DOI:** 10.1001/jamanetworkopen.2019.17571

**Published:** 2019-12-13

**Authors:** Sidra Goldman-Mellor, Mark Olfson, Cristina Lidon-Moyano, Michael Schoenbaum

**Affiliations:** 1 Department of Public Health, School of Social Sciences, Humanities, and Arts, University of California, Merced; 2 Department of Psychiatry, Columbia University, New York, New York; 3 Division of Services and Intervention Research, National Institute of Mental Health, Bethesda, Maryland

## Abstract

**Importance:**

Emergency departments (EDs) have the potential to play a pivotal role in suicide risk detection and prevention, yet little is known about the profile of risk of suicide after ED visits in the United States.

**Objectives:**

To examine 1-year incidence of suicide and other mortality among ED patients who presented with nonfatal deliberate self-harm, suicidal ideation, or any other chief concern, and to examine sociodemographic and clinical factors associated with suicide mortality risk.

**Design, Setting, and Participants:**

This retrospective cohort study included statewide, all-payer, longitudinally linked ED patient records and mortality data from all California residents who presented to a California-licensed ED at least 1 time from January 1, 2009, to December 31, 2011, with deliberate self-harm, suicidal ideation but not self-harm, or neither (a 5% random sample). Age-, sex-, and race/ethnicity-adjusted standardized mortality ratios (SMRs) for suicide and other manners or causes of death were determined for each patient group using statewide mortality data. Data were analyzed from January 10 to July 18, 2019.

**Main Outcomes and Measures:**

Suicide and other manners or causes of death were ascertained using *International Statistical Classification of Diseases and Related Health Problems, Tenth Revision* codes. Suicide rate and all mortality rates were measured per 100 000 person-years.

**Results:**

Among 648 646 individuals (mean [SD] age, 43.8 [20.6] years; 350 687 [54.1%] women) who visited an ED in California from 2009 to 2011, the rates of suicide deaths per 100 000 person-years in the year after index ED presentation were 693.4 deaths among 83 507 individuals presenting with deliberate self-harm (SMR, 56.8; 95% CI, 52.1-61.4), 384.5 deaths among 67 379 individuals presenting with suicidal ideation but not self-harm (SMR, 31.4; 95% CI, 27.5-35.2), and 23.4 deaths among 497 760 reference patients (SMR, 1.9; 95% CI, 1.6-2.3). Compared with the demographically matched general population, the rates of nonsuicide external-cause mortality were also increased among patients with self-harm (SMR, 14.2; 95% CI, 12.9-15.5), patients with suicidal ideation (SMR, 11.8; 95% CI, 10.6-13.0), and reference patients (SMR, 2.2; 95% CI, 2.0-2.3). In all 3 groups, the rates of suicide mortality per 100 000 person-years were higher among men (deliberate self-harm: 1011.1 deaths; suicidal ideation: 539.8 deaths; reference: 36.6 deaths), people 65 years or older (deliberate self-harm: 1919.5 deaths; suicidal ideation: 691.2 deaths; reference: 28.6 deaths), and non-Hispanic white patients (deliberate self-harm: 914.1 deaths; suicidal ideation: 511.6 deaths; reference: 33.8 deaths) than among their respective referent groups. Other sociodemographic factors and clinical diagnoses were associated with striking differences in suicide rates, but these patterns were heterogeneous across patient groups.

**Conclusions and Relevance:**

These findings suggest that ED patients with deliberate self-harm or suicidal ideation are associated with substantially increased risk of suicide and other mortality during the year after ED presentation. The process of planning for ED discharge may present opportunities to help ensure safe transitions to continuing outpatient mental health care and to consider broader risk for unintentional injury and other causes of premature mortality.

## Key Points

**Question:**

Is emergency department presentation associated with 1-year incidence of suicide and other mortality, and does the suicide rate vary by patient clinical and sociodemographic characteristics?

**Findings:**

In this cohort study including 648 646 patients who presented to California emergency departments, compared with the demographically matched general population, suicide mortality was 56.8-fold higher among patients presenting with deliberate self-harm, 31.4-fold higher among patients presenting with suicidal ideation, and 1.9-fold higher among patients presenting with any other chief concern; risk of other mortality was also increased. Sociodemographic and clinical factors associated with suicide risk varied by patient group.

**Meaning:**

These findings suggest that broad implementation of suicide risk screening and intervention is needed in emergency department settings, and the scope of interventions should also consider suicidal individuals’ risk for unintentional injury and other premature mortality.

## Teaser

This cohort study examines the association of suicide and other mortality risk within 1 year of emergency department presentation among patients presenting with deliberate self-harm, suicidal ideation, or any other chief concern.

## Introduction

Each year in the United States, more than 500 000 people present to emergency departments (EDs) with deliberate self-harm, and many more people present with suicidal ideation.^1^ Because self-harm and suicidal ideation are associated with increased risk of death by suicide,^2^ EDs offer potentially important settings in which to deliver frontline suicide prevention services and to help ensure patient safety by coordinating transfers or referrals to the appropriate level of continuing care. Practice guidelines for management of deliberate self-harm recommend clinical assessment of ongoing risk of self-harm and suicide.^3,4^ Although effective ED-based interventions for patients at high risk of suicide have been developed, they are not yet widely used in community practice.^5,6^

Little is known about the profile of risk of suicide after ED visits for self-harm or suicidal ideation in the United States. Past studies using large ED databases, such as the Healthcare Cost and Utilization Project data, cannot be linked to mortality records,^7^ and mortality-linkage studies using samples of US military patients, outpatients, and psychiatric inpatients differ considerably from ED patients in demographic characteristics, clinical severity, and treatment protocols.^8,9^ Other studies have examined mortality among single-payer (eg, Medicaid) patient pools, but findings from such specialized subpopulations are not generalizable.^10^ Although a few studies have examined suicide among US-based general-population ED patients, they all had limited statistical power, particularly to examine variability in patient suicide rates according to demographic and clinical characteristics, and none examined nonsuicide mortality, to our knowledge.^11,12^

To help address this gap in knowledge, this study examines longitudinal patterns of mortality among residents of California who visited an ED. We focus on 3 patient groups: those presenting with deliberate self-harm (with or without suicidal ideation), those presenting with suicidal ideation (without deliberate self-harm), and those presenting without deliberate self-harm or suicidal ideation, representing a random 5% sample, as a reference population. To our knowledge, this analysis represents the first US population-based study of suicide and other mortality after ED presentation with deliberate self-harm or suicidal ideation. To inform clinical practice, the study also advances prior work by examining how clinical correlates and sociodemographic variables of interest within each patient group are associated with suicide risk. Prior to performing these analyses, we hypothesized that both in the presence and absence of diagnosed mental disorders, a gradient in suicide risk would be observed, with patients presenting with deliberate self-harm having greater risk than those with suicidal ideation, who in turn would have greater risk than patients with neither self-harm nor suicidal ideation.

## Methods

This study was approved by the institutional review boards of the California Health and Human Services Agency and the University of California, Merced. Because the study comprised analyses of secondary data, a waiver of informed consent was granted by the California Health and Human Services Agency institutional review board. This study was prepared in accordance with the standards set forth by the Strengthening the Reporting of Observational Studies in Epidemiology (STROBE) reporting guideline.

### Data Sources

Discharge data were obtained from the California Office of Statewide Health Planning and Development on all visits to all California-licensed EDs from January 1, 2009, to December 31, 2011 by individuals 10 years or older with a California residential zip code. The Office of Statewide Health Planning and Development also provided information on all individuals in this ED cohort to the California Department of Public Health Vital Records, which assessed vital status in California death records and provided information on date and underlying cause or manner of death for all matching decedents who died from January 1, 2009, to December 31, 2012, excluding individuals who died out of state, who made up less than 1% of the total population of decedents. All data obtained and used by the study team were deidentified and reflect the most recent year that comprehensive death linkages were available. Linkages were implemented based on the patient’s Social Security number, self-reported sex, birthdate, self-reported race/ethnicity, and zip code of residence; patient ED records lacking a valid Social Security number (approximately 12%) were not eligible for linkage and were excluded from analysis.

The cohort was partitioned into 3 nonoverlapping groups: (1) patients with deliberate self-harm, defined as those with at least 1 ED visit during the study period that included an *International Classification of Diseases, Ninth Revision, Clinical Modification* (*ICD-9-CM*)^13^ external cause-of-injury code (E-code) of E950.0-958, in any diagnostic position; (2) patients with suicidal ideation, defined as those with at least 1 ED visit during the period that included an *ICD-9-CM* diagnosis of V62.84 in any diagnostic position but not with a diagnosis for deliberate self-harm; and (3) a 5% random sample of all other patients (hereafter, *reference patients*). California has mandated 100% reporting of E-codes since 1990. For patients with self-harm or suicidal ideation, their first qualifying ED visit during the study period was defined as their index visit. For reference patients, their first ED visit during the period was defined as the index visit. The analyses were limited to index visits.

For visits resulting in patient discharge or transfer to another facility, the index date was the date of ED presentation. For visits that resulted in a same-hospital admission, the index date was the date of hospital discharge from the associated hospital admission. Patients who died on the date of the index visit or whose index visit disposition was coded as death were excluded from follow-up analyses.

We obtained data on death by cause for California overall in 2009 to 2012 from the Centers for Disease Control and Prevention’s Web-based Injury Statistics Query and Reporting System^14^ and Wide-ranging Online Data for Epidemiologic Research^15^ systems.

### Measures

Our primary outcome was suicide within 1 year of the index date. Suicide was defined as any death with *International Statistical Classification of Diseases and Related Health Problems, Tenth Revision* (*ICD-10*)^16^ codes X60-X84, Y87.0, or U03 as the immediate cause of death. Secondary outcomes included deaths by unintentional injury and other causes, based on corresponding *ICD-10* codes.^16^

Patient demographic factors of interest included sex (male or female), age group (10-24, 25-44, 45-64, ≥65 years), race/ethnicity (collapsed into non-Hispanic white, non-Hispanic black, Hispanic, Asian or Pacific Islander, or other), and insurance status. Insurance was classified as private (eg, employment-based coverage), Medi-Cal (ie, California’s Medicaid program, which provides low-cost or free health coverage to eligible residents with limited income), Medicare (federal health insurance for people aged ≥65 years or who have disabilities), or self-pay or other. Patients’ urbanicity was defined via zip code of residence using the US Department of Agriculture’s Rural-Urban Commuting Areas 2010 geographic taxonomy, version 3.10 (collapsed into metropolitan, micropolitan, or small town or rural).^17^

Patient clinical factors of interest included comorbid clinical diagnoses recorded at the index ED visit and, for patients with deliberate self-harm, method of index self-harm injury. Comorbid diagnoses were ascertained using *ICD-9-CM* codes and Clinical Classification Software (CCS) groupings, which aggregate *ICD-9-CM* diagnoses into discrete, clinically meaningful categories. We focused on psychiatric diagnoses known to be associated with suicide risk: depression (*ICD-9* codes 296.2-296.3, 296.82, 298.0, 298.82, 300.4, and 311), bipolar disorder (*ICD-9* codes 296.0-296.1 and 296.4-296.8),^13^ anxiety disorder (CCS code 651), psychotic disorder (CCS code 659), alcohol-related disorder (CCS code 660), and drug-related disorder (CCS code 661); we also included suicidal ideation for patients with deliberate self-harm. We also calculated the Elixhauser Comorbidity Index, a claims-based scale that counts up to 31 categories of comorbid physical and psychological conditions and has good predictive validity for short-term mortality.^18^

Injury method among patients with deliberate self-harm was categorized according to the most common methods observed among California ED patients: poisoning, cutting or piercing, hanging, jumping, firearm, and all others.^19^ The *ICD-9-CM* codes^13^ used to define this variable are shown in the eTable in the Supplement.

### Statistical Analysis

We calculated crude mortality rates per 100 000 person-years of follow-up for suicide and other manners or causes of death in the year after the index ED visit for self-harm, suicidal ideation, and reference groups. We additionally calculated crude suicide mortality rates per 100 000 person-years of follow-up within each group for the demographic and clinical categories of interest. Individuals whose information was not linked to California mortality records from 365 days after the index date were presumed alive for this period. Decedents were treated as censored on their death date; patients who died more than 365 days after their index visit were assumed alive at the 1-year point.

For our second aim, we restricted analyses to each patient group and modeled incidence rate ratios estimating associations between each variable and rate of suicide by end of follow-up. We opted to estimate risk ratios (RRs) rather than hazard ratios because several variables did not meet proportional-hazards assumptions required for Cox regression. All models included an offset term consisting of person-time observed for each patient during follow-up. We first estimated bivariate unadjusted RRs. We then fit adjusted models that controlled for patient age, sex, and race/ethnicity, as these factors are important independent factors associated with suicide risk.

As a frame of reference, we calculated annualized standardized mortality ratios (SMRs), defined as the ratio of the observed numbers of deaths in our ED cohorts to expected deaths. The rates of expected deaths in California for 2009 to 2012 for suicide and other manners or causes of death were calculated using the Centers for Disease Control and Prevention Web-based Injury Statistics Query and Reporting System^14^ and Wide-ranging Online Data for Epidemiologic Research^15^ mortality databases, standardized to the distribution of sex, age category, and race/ethnicity category of the respective groups in our ED cohorts. For each respective ED cohort, standardization was implemented by multiplying the proportion of patients in that cohort’s age-, sex-, and race-specific strata by the age-, sex-, and race-specific mortality rates observed statewide.

Statistical analyses were conducted using Stata version 14.0 (StataCorp). *P* values were 2-tailed, and statistical significance was set at an α of .05. Data were analyzed from January 10 to July 18, 2019.

## Results

### Cohort Characteristics

The cohort included 648 646 individuals (mean [SD] age, 43.8 [20.6] years; 350 687 [54.1%] women) who visited an ED in California from 2009 to 2011. There were 83 507 patients with deliberate self-harm (mean [SD] age, 35.3 [15.8] years; 49 120 [58.8%] women) (Table 1), 67 379 patients with suicidal ideation (mean [SD] age, 40.4 [16.6] years; 32 825 [51.3%] women) (Table 2), and 497 760 reference patients (mean [SD] age, 45.7 [21.4] years; 268 742 [54.0%] women) (Table 3) who had nonfatal index events and were included in follow-up analyses. Patients with self-harm or suicidal ideation were younger than reference patients, and they were more likely to be of non-Hispanic white race/ethnicity (deliberate self-harm: 48 558 [58.2%] patients; suicidal ideation: 38 292 [56.8%]; reference: 247 087 [49.6%] patients).

**Table 1.** Suicide Within 1 Year Among California Emergency Department Patients Presenting With Deliberate Self-harm Stratified by Patient Demographic and Clinical Characteristics

Characteristic	No. (%)	Suicides, No.	Suicide Rate, Per 100 000 Person-Years	Suicide Rate Ratio (95% CI)	
				Unadjusted^a^	Adjusted^b^
Total patients	83 507	570	693.4		
Sex					
Women	49 120 (58.8)	230	473.5	1 [Reference]	NA
Men	34 387 (41.2)	340	1011.1	2.14 (1.81-2.53)	NA
Age, y					
10-24	27 218 (32.6)	60	221.3	1 [Reference]	NA
25-44	32 043 (38.4)	215	678.6	3.07 (2.30-4.08)	NA
45-64	20 762 (24.9)	235	1159.1	5.24 (3.95-6.96)	NA
≥65	3484 (4.2)	60	1919.5	8.68 (6.06-12.42)	NA
Race/ethnicity					
Non-Hispanic white	48 558 (58.2)	435	914.1	1 [Reference]	NA
Non-Hispanic black	7574 (9.1)	17	226.5	0.25 (0.15-0.40)	NA
Hispanic	19 290 (23.1)	73	381.8	0.42 (0.33-0.54)	NA
Asian or Pacific Islander	3473 (4.2)	20	584.3	0.64 (0.41-1.00)	NA
Other	4612 (5.5)	25	547.6	0.60 (0.40-0.90)	NA
Insurance or payer					
Private	29 616 (35.5)	221	755.3	1 [Reference]	1 [Reference]
Medicaid	24 344 (29.2)	118	490.4	0.65 (0.52-0.81)	0.72 (0.57-0.91)
Medicare	9091 (10.9)	115	1332.4	1.76 (1.41-2.21)	0.98 (0.77-1.25)
Self-pay or other	20 416 (24.5)	116	574.0	0.76 (0.61-0.95)	0.72 (0.57-0.91)
Rurality of residence					
Metropolitan	76 213 (91.3)	507	675.6	1 [Reference]	1 [Reference]
Micropolitan	5152 (6.2)	45	889.9	1.32 (0.97-1.79)	1.19 (0.87-1.62)
Rural or small town	2142 (2.6)	18	856.8	1.27 (0.79-2.03)	1.10 (0.69-1.77)
Comorbid diagnosis					
Depressive disorder	37 241 (41.9)	268	778.6	1.23 (1.05-1.45)	1.14 (0.96-1.35)
Bipolar disorder	9288 (11.1)	92	1006.1	1.54 (1.23-1.92)	1.45 (1.16-1.81)
Anxiety disorder	9392 (11.3)	89	966.5	1.47 (1.17-1.84)	1.44 (1.15-1.81)
Psychotic disorder	4710 (5.7)	51	1107.8	1.66 (1.24-2.21)	1.36 (1.02-1.82)
Alcohol disorder	15 902 (19.0)	123	786.8	1.17 (0.96-1.43)	0.92 (0.75-1.13)
Drug disorder	16 021 (19.2)	129	819.7	1.24 (1.02-1.50)	1.14 (0.93-1.39)
Suicidal ideation	9396 (11.3)	72	777.2	1.14 (0.89-1.46)	1.12 (0.87-1.43)
Elixhauser Comorbidity Index score					
0	26 172 (31.3)	97	373.3	1 [Reference]	1 [Reference]
1	26 011 (31.2)	139	539.6	1.40 (1.33-1.47)	1.23 (1.16-1.30)
2	15 339 (18.4)	122	808.6		
3	8061 (9.7)	89	1131.3		
4	4145 (5.0)	49	1224.5		
≥5	3779 (4.5)	74	2113.7		
Method of index self-harm					
Poisoning	57 364 (68.7)	409	724.3	1 [Reference]	1 [Reference]
Cutting or piercing	17 806 (21.3)	90	511.1	0.71 (0.56-0.89)	0.81 (0.65-1.03)
Hanging	1483 (1.8)	39	2723.8	3.76 (2.70-5.24)	3.73 (2.66-5.24)
Jumping	555 (0.7)	<11^c^	556.7	0.77 (0.25-2.39)	0.71 (0.23-2.22)
Firearm	361 (0.4)	15	4444.1	6.13 (3.64-10.3)	4.04 (2.39-6.84)
Other	5938 (7.1)	14	240.5	0.33 (0.20-.57)	0.36 (0.21-0.63)

Abbreviation: NA, not applicable.

^a^ Bivariate models.

^b^ Models controlled for patient age at index visit, sex, and race/ethnicity.

^c^ Exact cell counts suppressed to protect patient confidentiality.

**Table 2.** Suicide Within 1 Year Among California Emergency Department Patients Presenting With Suicidal Ideation Stratified by Patient Demographic and Clinical Characteristics

Characteristic	No. (%)	Suicides, No.	Suicide Rate Per 100 000 Person-Years	Suicide Rate Ratio (95% CI)	
				Unadjusted^a^	Adjusted^b^
Total patients	67 379	254	384.5		
Sex					
Women	32 825 (51.3)	72	222.7	1 [Reference]	NA
Men	34 554 (48.7)	182	539.8	2.42 (1.85-3.19)	NA
Age, y					
10-24	14 438 (21.4)	34	236.4	1 [Reference]	NA
25-44	24 650 (36.6)	72	294.6	1.25 (0.83-1.87)	NA
45-64	23 450 (34.8)	118	515.5	2.18 (1.49-3.20)	NA
≥65	4841 (7.2)	30	691.2	2.92 (1.79-4.78)	NA
Race/ethnicity					
Non-Hispanic white	38 292 (56.8)	191	511.6	1 [Reference]	NA
Non-Hispanic black	8942 (13.3)	11	124.4	0.24 (0.13-0.45)	NA
Hispanic	14 136 (21.0)	29	207.7	0.41 (0.28-0.60)	NA
Asian or Pacific Islander	2605 (3.9)	14	549.8	1.08 (0.62-1.85)	NA
Other	3404 (5.1)	<11^c^	267.1	0.52 (0.27-1.02)	NA
Insurance or payer					
Private	15 378 (31.2)	100	483.9	1 [Reference]	1 [Reference]
Medicaid	19 224 (28.6)	49	258.6	0.53 (0.38-0.75)	0.62 (0.44-0.88)
Medicare	11 720 (17.4)	47	421.8	0.87 (0.62-1.23	0.61 (0.42-0.88)
Self-pay or other	15 378 (22.9)	58	381.0	0.79 (0.57-1.09)	0.77 (0.56-1.08)
Rurality of residence					
Metropolitan	63 536 (94.3)	236	378.8	1 [Reference]	1 [Reference]
Micropolitan	2702 (4.0)	11	416.5	1.10 (0.60-2.01)	0.98 (0.53-1.80)
Rural or small town	1141 (1.7)	<11^c^	626.5	1.65 (0.78-3.51)	1.44 (0.68-3.04)
Comorbid diagnosis					
Depressive disorder	17 577 (49.0)	145	449.3	1.39 (1.09-1.79)	1.35 (1.05-1.73)
Bipolar disorder	10 081 (15.0)	33	332.1	0.84 (0.58-1.22)	0.86 (0.60-1.24)
Anxiety disorder	6831 (10.1)	34	506.6	1.37 (0.95-1.96)	1.33 (0.92-1.82)
Psychotic disorder	4237 (6.3)	<11^c^	217.8	0.55 (0.28-1.07)	0.54 (0.28-1.05)
Alcohol use disorder	11 348 (16.8)	56	505.1	1.40 (1.04-1.89)	1.15 (0.85-1.56)
Drug use disorder	12 951 (19.2)	49	384.3	1.00 (0.73-1.37)	1.03 (0.75-1.42)
Elixhauser Comorbidity Index score					
0	22 939 (34.0)	89	391.5	1 [Reference]	1 [Reference]
1	19 615 (29.1)	78	402.3	0.99 (0.90-1.08)	0.90 (0.82-0.99)
2	11 723 (17.4)	42	364.2		
3	6354 (9.4)	19	308.1		
4	3323 (4.9)	13	410.3		
≥5	3425 (5.1)	13	423.4		

Abbreviation: NA, not applicable.

^a^ Bivariate models.

^b^ Models controlled for patient age at index visit, sex, and race/ethnicity.

^c^ Exact cell counts suppressed to protect patient confidentiality.

**Table 3.** Suicide Within 1 Year Among California Emergency Department Patients Presenting With Any Other Chief Concern Stratified by Patient Demographic and Clinical Characteristics

Characteristic	No. (%)	Suicides, No.	Suicide Rate Per 100 000 Person-Years	Suicide Rate Ratio (95% CI)	
				Unadjusted^a^	Adjusted^b^
Total patients	497 760	114	23.4		
Sex					
Women	268 742 (54.0)	32	12.2	1 [Reference]	NA
Men	229 018 (46.0)	82	36.6	3.02 (2.01-4.54)	NA
Age, y					
10-24	100 337 (20.2)	15	15.0	1 [Reference]	NA
25-44	150 395 (30.2)	30	20.0	1.34 (0.72-2.48)	NA
45-64	141 197 (28.4)	41	29.4	1.97 (1.09-3.55)	NA
≥65	105 831 (21.3)	28	28.6	1.91 (1.02-3.58)	NA
Race/ethnicity					
Non-Hispanic white	247 087 (49.6)	81	33.8	1 [Reference]	NA
Non-Hispanic black	47 328 (9.5)	<11^c^	10.7	0.32 (0.13-0.78)	NA
Hispanic	135 650 (27.3)	18	13.4	0.40 (0.24-0.66)	NA
Asian or Pacific Islander	36 987 (7.4)	<11^c^	19.4	0.57 (0.27-1.24)	NA
Other	30 708 (6.2)	<11^c^	9.9	0.29 (0.09-0.93)	NA
Insurance or payer					
Private	233 694 (47.0)	47	20.4	1 [Reference]	1 [Reference]
Medicaid	84 181 (16.9)	16	19.2	0.94 (0.54-1.66)	1.19 (0.66-2.14)
Medicare	81 075 (16.3)	23	30.8	1.52 (0.92-2.50)	1.15 (0.62-2.14)
Self-pay or other	98 310 (19.8)	28	28.6	1.41 (0.88-2.25)	1.43 (0.87-2.35)
Rurality of residence					
Metropolitan	462 421 (92.9)	104	23.0	1 [Reference]	NA^d^
Micropolitan	24 338 (4.9)	<11^c^	42.0	1.83 (0.96-3.49)	NA^d^
Rural or small town	10 997 (2.2)	0	0	NA^d^	NA^d^
Comorbid diagnosis					
Depressive disorder	14 178 (3.1)	10	22.0	3.15 (1.65-6.02)	3.01 (1.56-5.84)
Bipolar disorder	2747 (0.6)	<11^c^	69.2	8.35 (3.40-20.46)	7.33 (2.98-18.02)
Anxiety disorder	11 047 (2.2)	<11^c^	37.2	1.61 (0.59-4.37)	1.27 (0.46-3.54)
Psychotic disorder	2382 (0.5)	<11^c^	44.9	1.93 (0.27-13.8))0	1.04 (0.15-7.37)
Alcohol disorder	10 126 (2.0)	14	144.5	6.90 (3.94-12.07)	4.13 (2.17-7.84)
Drug disorder	7081 (1.4)	<11^c^	101.0	4.54 (2.11-9.74)	2.23 (0.93-5.33)
Elixhauser Comorbidity Index score					
0	324 343 (65.2)	61	18.9	1 [Reference]	1 [Reference]
1	83 986 (16.9)	28	34.0	1.18 (1.05-1.33)	1.12 (0.97-1.29)
2	41 428 (8.3)	<11^c^	20.1		
3	21 363 (4.3)	<11^c^	35.4		
4	12 140 (2.4)	<11^c^	65.0		
≥5	14 500 (2.9)	<11^c^	25.2		

Abbreviation: NA, not applicable.

^a^ Bivariate models.

^b^ Models controlled for patient age at index visit, gender, and race/ethnicity.

^c^ Exact cell counts suppressed to protect patient confidentiality.

^d^ Risk ratio was undefined, as the numerator value for the rural group was 0.

An additional 4040 individuals died on the date of the index event and were excluded from analysis, including 820 patients with deliberate self-harm (73.9% listed as dying by suicide), 104 patients with suicidal ideation (3.9% listed as dying by suicide), and 3116 reference patients (0.3% listed as dying by suicide). Of 15 patients with deliberate self-harm who were discharged alive but died on their index date, 13 died by suicide. No patients in the other groups who were discharged alive died by suicide on their index date.

Among all patients, the cumulative probability of suicide in the first year after discharge was highest among patients with self-harm, then patients with suicidal ideation, then reference patients (eFigure in the Supplement).

### Patterns and Associations of Suicide After the Index Date

In patients whose index ED visit involved deliberate self-harm (Table 1), the suicide rate in the subsequent year was 693.4 deaths per 100 000 person-years and the SMR was 56.8 (95% CI, 52.1-61.4) (Table 4). If deaths among patients with self-harm who were discharged from the ED alive but died by suicide on their index ED date were included, this rate would be 709.2 deaths per 100 000 person-years. This overall rate masked substantial variability among patients with different demographic and clinical characteristics. Consistent with previous research,^14^ men (1011.1 deaths per 100 000 person-years) and patients 65 years and older (1919.5 deaths per 100 000 person-years) had markedly higher rates of suicide compared with women (473.5 deaths per 100 000 person-years) and patients aged 10 to 24 years (221.3 deaths per 100 000 person-years). Patients who were non-Hispanic black (226.5 deaths per 100 000 person-years) had substantially lower rates compared with patients who were non-Hispanic white (914.1 deaths per 100 000 person-years). Suicide rates among patients with Medicaid coverage (490.4 deaths per 100 000 person-years) or self-pay or other health care payer (574.0 deaths per 100 000 person-years) were also lower than rates among privately insured patients (755.3 deaths per 100 000 person-years). Rurality of residence was not significantly associated with suicide risk.

**Table 4.** Mortality Within 1 Year Among California ED Patients Presenting With Deliberate Self-Harm, Suicidal Ideation, or Any Other Chief Concern Stratified by Manner or Cause of Death

Manner of Death	Deliberate Self-harm Group (n = 83 507)	Suicidal Ideation Group (n = 67 379)	Reference Group (n = 497 760)^a^
Suicide			
ED cohort, rate per 100 000 person-years	693.4	384.5	23.4
California population, annual rate per 100 000 person-years^b^	12.3	14.7	13.3
SMR (95% CI)	56.8 (52.1-61.4)	31.4 (27.5-35.2)	1.9 (1.6-2.3)
Unintentional injury			
ED cohort, rate per 100 000 person-years	487.8	498.1	89.5
California population, annual rate per 100 000 person-years^b^	30.7	37.5	40.0
SMR (95% CI)	15.6 (14.1-17.2)	13.0 (11.6-14.4)	2.2 (2.0-2.4)
Homicide or legal intervention			
ED cohort, rate per 100 000 person-years	21.9	27.3	11.3
California population, annual rate per 100 000 person-years^b^	6.2	7.5	6.1
SMR (95% CI)	3.5 (1.9-5.1)	3.6 (1.9-5.2)	1.8 (1.3-2.3)
Undetermined intent			
ED cohort, rate per 100 000 person-years	40.1	33.3	2.9
California population, annual rate per 100 000 person-years^b^	1.2	1.4	1.2
SMR (95% CI)	31.7 (20.9-42.6)	23.3 (13.6-33.1)	2.3 (1.1-3.4)
Natural			
ED cohort, rate per 100 000 person-years	1248.1	2348.1	3226.7
California population, annual rate per 100 000 person-years^b^	319.9	504.5	993.1
SMR (95% CI)	3.8 (3.6-4.1)	4.6 (4.3-4.8)	3.2 (3.1-3.2)
All-cause			
ED cohort, rate per 100 000 person-years	2491.4	3291.2	3353.7
California population, annual rate per 100 000 person-years^b^	370.3	565.6	1053.7
SMR (95% CI)	6.6 (6.3-6.9)	5.7 (5.5-5.9)	3.1 (3.1-3.2)
Nonsuicide external cause^c^			
ED cohort, rate per 100 000 person-years	549.9	558.6	103.6
California population, annual rate per 100 000 person-years^b^	38.1	46.5	47.3
SMR (95% CI)	14.2 (12.9-15.5)	11.8 (10.6-13.0)	2.2 (2.0-2.3)

Abbreviations: ED, emergency department; SMR, standardized mortality ratio.

^a^ Based on 5% random sample.

^b^ Calculated from California data for 2009 to 2012 from CDC WISQARS (external causes) and WONDER (all-cause), standardized to match the distribution of sex, age category, and race/ethnicity category of the corresponding group from the ED cohort.

^c^ Including unintentional injury, homicide or legal intervention, and undetermined intent.

Among patients with self-harm, several comorbid clinical diagnoses (ie, depression, bipolar, anxiety, psychotic, and drug use disorders) were associated with elevated suicide risk in unadjusted models, but some associations were reduced to nonsignificance in the adjusted models. Patients with deliberate self-harm with a comorbid diagnosis of bipolar disorder (adjusted RR, 1.45; 95% CI, 1.16-1.81), anxiety disorder (adjusted RR, 1.44; 95% CI, 1.15-1.81), or psychotic disorder (adjusted RR, 1.36; 95% CI, 1.02-1.82) remained statistically significantly more likely to die by suicide than patients without those diagnoses. Among patients with self-harm, higher Elixhauser Comorbidity Index scores were associated with increased suicide risk in a dose-response manner (adjusted RR, 1.23; 95% CI, 1.16-1.30). Firearm injury at index visit was also associated with increased risk of suicide death (adjusted RR, 4.04; 95% CI, 2.39-6.84). Patients whose nonfatal index self-harm event involved a firearm had a suicide incidence rate of 4444.1 deaths per 100 000 person-years, far higher than any other patient group.

In the suicidal ideation group (Table 2), the suicide rate in the year after index ED visit was 384.5 deaths per 100 000 person-years and the SMR was 31.4 (95% CI, 27.5-35.2) (Table 4). As with patients with self-harm, men (539.8 deaths per 100 000 person-years) and patients 65 years or older (691.2 deaths per 100 000 person-years) had higher rates of suicide compared with women (222.7 deaths per 100 000 person-years) or patients aged 10 to 24 years (236.4 deaths per 100 000 person-years), and non-Hispanic black patients (124.4 deaths per 100 000 person-years) and Hispanic patients (207.7 deaths per 100 000 person-years) had lower suicide rates than non-Hispanic white patients (511.6 deaths per 100 000 person-years). Medicaid insurance was also associated with a lower suicide rate (258.6 deaths per 100 000 person-years) than private insurance (483.9 deaths per 100 000 person-years). After covariate adjustment, comorbid diagnosis with depression was associated with increased suicide risk (adjusted RR, 1.35; 95% CI, 1.05-1.73), although increasing Elixhauser Comorbidity Index scores were associated with lower suicide risk in the suicidal ideation cohort (adjust RR, 0.90; 95% CI, 0.82-0.99). No other demographic or clinical characteristics were associated with suicide risk in adjusted models.

Among reference patients (Table 3), the suicide rate in the year after index ED visit was 23.4 deaths per 100 000 person-years, substantially lower than in the other groups, although still almost 2-fold that of the matched California population (SMR, 1.9; 95% CI, 1.6-2.3) (Table 4). As in the other groups, men (36.6 deaths per 100 000 person-years) and patients 65 years and older (28.6 deaths per 100 000 person-years) had higher rates of suicide than women (12.2 deaths per 100 000 person-years) and patients aged 10 to 24 years (15.0 deaths per 100 000 person-years); and patients who were non-Hispanic black (10.7 deaths per 100 000 person-years) or Hispanic (13.4 deaths per 100 000 person-years) again had lower suicide rates than non-Hispanic white patients (33.8 deaths per 100 000 person-years). However, insurance status was not associated with suicide risk in the reference group. In adjusted analyses, reference patients with comorbid diagnosed depression (adjusted RR, 3.01; 95% CI, 1.56-5.84), bipolar disorder (adjusted RR, 7.33; 95% CI, 2.98-18.02), and alcohol use disorder (adjusted RR, 4.13; 95% CI, 2.17-7.84) had increased suicide risk, but other clinical diagnoses, including Elixhauser Comorbidity Index, were not associated with increased risk of suicide.

The Figure shows suicide rates among each patient group according to presence of the comorbid psychiatric diagnoses. Despite variation in levels of suicide risk across diagnostic groups, patients with deliberate self-harm were consistently the highest risk group, then patients with suicidal ideation, then reference patients.

**Figure.**
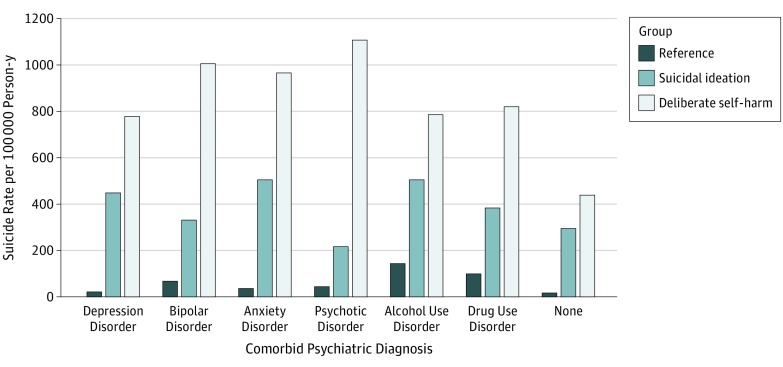
Suicide Rates Within 1 Year of Emergency Department Visit Stratified by Comorbid Psychiatric Diagnosis and Patient Group

### Other Causes of Death

Beyond our primary outcome of suicide within 1 year of ED presentation, we also examined rates of mortality by other causes (Table 4). In the year after index ED visit and compared with the demographically matched general population, the rates of nonsuicide external-cause mortality were disproportionately high among patients with self-harm (SMR, 14.2.; 95% CI, 12.9-15.5), patients with suicidal ideation (SMR, 11.8; 95% CI, 10.6-13.0), and reference patients (SMR, 2.2; 95% CI, 2.0-2.3). Overdoses composed 72% of all unintentional deaths in the self-harm group and 61% of deaths in the suicidal ideation group. Furthermore, the natural-cause mortality rate was also markedly increased among patients with self-harm (SMR, 3.8; 95% CI, 3.6-4.1), patients with suicidal ideation (SMR, 4.6; 95% CI, 4.3-4.8), and reference patients (SMR, 3.2; 95% CI, 3.1-3.2) (Table 4).

## Discussion

This cohort study is the first US population-based study to examine 12-month suicide rates after an index ED visit, to our knowledge. In the state of California from 2009 to 2012, we found substantially increased and enduring risk of suicide death among patients who presented to an ED with deliberate self-harm or suicidal ideation. The suicide rate of the self-harm sample was 2-fold that reported from a self-harm sample at a university-affiliated ED.^11^ These findings reinforce the rationale for providing ED-based within-encounter and postdischarge interventions, including safety planning, follow-up communications, mobile crisis team linkage, and text reminders, that have evidence for effectiveness but are not yet widely used.^5,6,20^

Even ED patients with no self-harm or suicidal ideation experienced postindex suicide rates almost 2-fold that of the corresponding California population. Together, these findings reinforce the potential value of routinely screening all ED patients for suicide risk, an approach that has been found to increase the number of ED patients identified as warranting treatment for suicide risk by approximately 2-fold,^21^ but which is also not yet widespread.

Patients in both the self-harm and ideation groups also had markedly increased risk for other external cause–mortality, particularly via unintentional injury. Such overlap between suicide and unintentional injury mortality risk has been observed in other settings.^22^ This may reflect limitations in determining intent of death, particularly for deaths via unintentional overdoses.^23^ In addition, there may be common etiologic factors, such as mental states, including impulsivity, inattentiveness, and hopelessness, across these causes of death. These patterns suggest that the scope of interventions for individuals with self-harm or suicidal behavior in ED settings should consider their broader risk for unintentional injury and other premature mortality.^22^

We found striking differences in suicide rates according to patients’ clinical diagnoses recorded at the index ED visit. These patterns were heterogeneous across patient subgroup, an observation that has not been previously reported, to our knowledge. For example, a clinical diagnosis of psychosis was associated with increased suicide risk among patients with deliberate self-harm but not among patients with suicidal ideation or reference patients. By contrast, alcohol use disorder in the absence of suicidal ideation or self-harm was associated with increased suicide risk among reference patients. In interpreting between-group variability, it is important to recognize that mental disorder diagnoses may be underrecorded in ED settings, and the extent of the underrecording likely varies with the presenting chief concern.^24^ As the Figure demonstrates, principal cohort membership (ie, deliberate self-harm, suicidal ideation, or reference) was associated with suicide risk across the clinical mental disorder diagnosis groups. The consistency of this finding, including among patients without clinical mental disorder diagnoses, suggests a need for suicide prevention efforts to consider factors beyond underlying mental health diagnoses.

Consistent with a 2017 study,^25^ patients with self-harm whose index ED visit was firearm related had the highest suicide rate of any subgroup studied. Along with a need for improved practices regarding limiting access to lethal means for individuals with suicide risk, including lethal means counseling, ED physicians should be aware that patients with self-harm who use high-lethality methods at a nonfatal event remain at highly increased risk for future death of suicide.

Within each patient group, sex, age, and race/ethnicity were associated with variability in suicide risk. These differences, which recreate in ED samples patterns in the general population, might be partially explained by variable access to and probability of using firearms, moral beliefs about suicide, and access to psychosocial resources and psychological treatment.^26,27^ Insurance status was also associated with suicide risk, albeit inconsistently. Notably, the suicide rates in this study for patients with self-harm who had Medicaid coverage closely resembled rates reported in a national study of patients with self-harm and Medicaid coverage,^25^ suggesting that this population experiences lower rates of suicide. This difference may be owing to better access to mental health care among patients with Medicaid coverage compared with individuals who are privately insured or differences in their sociocultural composition.^28^

### Limitations and Strengths

This study had several limitations. There are alternative ways to partition the ED cohort into subgroups; moreover, calculating person-level mortality rates likely underestimates visit-level mortality risk, as individuals who make multiple ED visits would presumably experience higher risk. This may be particularly true for individuals presenting with suicidal ideation, many of whom were classified into the self-harm group in this study. However, we believe alternative analytic strategies would be unlikely to change our main conclusions. Because the sociodemographic characteristics of California differ from those of other states^29^ and because California has a relatively low state suicide rate,^30^ the results may not be safely generalized to the entire United States. Mortality data for members of our ED cohort who died outside of California were not available for this study; we expect this artifact has a slight downward bias on the absolute mortality rates in our ED groups and on the comparison among the ED groups and California overall. Some suicide mortality occurring after the index date may have been due to the index self-harm event and therefore would be outside the reach of intervention initiated at the index ED visit. Additionally, *ICD-9* E-codes^13^ do not distinguish between deliberate self-harm with and without intent to die. Furthermore, all diagnostic codes used in the study were based on clinical judgment and were not subject to expert independent validation. External cause-of-injury coding in California is virtually complete (>95% of injury events are E-coded); nevertheless, 4.7% of injury or poisoning events did not have an associated E-code and could have been placed in the reference group. We did not attempt to validate the determined cause of death as recorded in our death certificate data. Furthermore, we lacked information about many potentially relevant patient risk factors, such as access to lethal means and engagement with outpatient care after ED discharge.

Our study also had important strengths. These included a census of the residents of California who visited EDs during a 3-year period, comprehensive coding of external cause of injury, which is not available in all states, and statewide ED discharge data linked to mortality records, which also may not be available in all states.

## Conclusions

The findings in this cohort study regarding the high risk of suicide after ED visits among patients with nonfatal deliberate harm or suicidal ideation underscores the clinical rationale for ensuring that these patients receive timely follow-up mental health care. This rationale is strengthened by evidence of low rates of outpatient mental health follow-up among patients with self-harm who are publicly or privately insured.^31^ Some promising interventions include universal screening,^21^ safety planning,^20^ active management of service transitions,^32^ and telephone follow-up and support.^5^ Moreover, conducting ongoing public health surveillance of the patterns and associations of suicide and other mortality among ED patients could inform health care quality improvement,^33^ help monitor progress toward national suicide prevention goals, and address issues of parity between mental and physical health care.

## Notes

Supplement. **eTable.** ICD-9-CM Codes Used to Define Method of Self-Harm Injury **eFigure.** Kaplan-Meier Cumulative Probabilities of Suicide for 1 Year of Follow-Up During 2009 to 2012 Among California Emergency Department Patients Stratified by Patient Group and Sex
