# Supplementary material for: Association of Suicide and Other Mortality With Emergency Department Presentation
Source: JAMA Netw Open. 2019 Dec 13;2(12):e1917571. doi: 10.1001/jamanetworkopen.2019.17571 (PMC6991205; doi:10.1001/jamanetworkopen.2019.17571)
Supplement: Supplement. — eTable. ICD-9-CM Codes Used to Define Method of Self-Harm Injury eFigure. Kaplan-Meier Cumulative Probabilities of Suicide for 1 Year of Follow-Up During 2009 to 2012 Among California Emergency Department Patients Stratified by Patient Group and Sex [file jamanetwopen-2-e1917571-s001.pdf]

Supplementary Online Content

Goldman-Mellor S, Olfson M, Lidon-Moyano C, Schoenbaum M. Association of suicide and other mortality with emergency department presentation. *JAMA Netw Open*. 2019;2(12):e1917571. doi:10.1001/jamanetworkopen.2019.17571

eTable. ICD-9-CM Codes Used to Define Method of Self-Harm Injury

eFigure. Kaplan-Meier Cumulative Probabilities of Suicide for 1 Year of Follow-Up During 2009 to 2012 Among California Emergency Department Patients Stratified by Patient Group and Sex

This supplementary material has been provided by the authors to give readers additional information about their work.

© 2019 Goldman-Mellor S et al. *JAMA Network Open.*

eTable. ICD-9-CM Codes Used to Define Method of Self-Harm Injury

**ICD-9-CM codes used to define method of self-harm injury.**	
**Category**	**ICD-9-CM codes**
Poisoning	E950.0-E952.x
Cut/pierce	E956.0-E956.x
Hanging/strangulation/suffocation	E953.0-E953.x
Jumping	E957.0-E957.x
Firearm	E955.0-E955.x
Other	E954.0-E954.x, E958.0-E958.x

© 2019 Goldman-Mellor S et al. *JAMA Network Open.*

eFigure. Kaplan-Meier Cumulative Probabilities of Suicide for 1 Year of Follow-Up During 2009 to 2012 Among California Emergency Department Patients Stratified by Patient Group and Sex

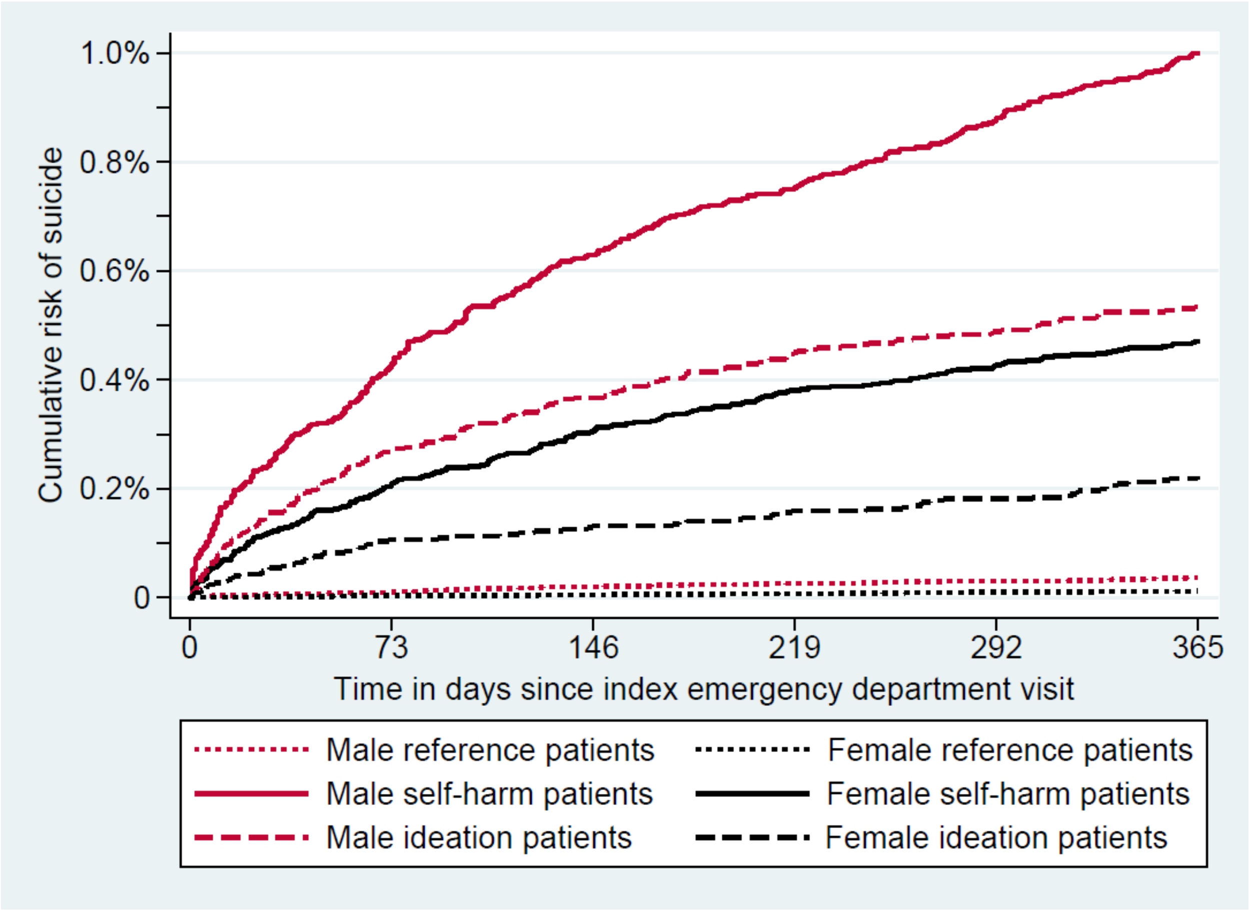

eFigure. Kaplan-Meier cumulative probabilities of suicide, by patient group and sex, for 365 days of follow-up during 2009-2012 among California emergency department patients. Log-rank tests comparing suicide rates among male vs. female patients were significant in all patient groups (all *p*<0.001).

© 2019 Goldman-Mellor S et al. *JAMA Network Open.*
